# Appropriateness of antimicrobial use among septic patients managed by the critical care response team: an opportunity for improvement through de-escalation

**DOI:** 10.1186/s13756-019-0609-0

**Published:** 2019-11-21

**Authors:** Saad M. Al-Qahtani, Henry Baffoe-Bonnie, Aiman El-Saed, Majid Alshamrani, Abdullah Algwizani, Ali Alaklabi, Khuloud AlJoudi, Nahlah Albaalharith, Azzam Mohammed, Sajid Hussain, Hanan H. Balkhy

**Affiliations:** 1 Intensive Care Medicine Department, King Abdulaziz Medical City, Riyadh, Saudi Arabia; 2 King Saud bin Abdulaziz University for Health Sciences, Riyadh, Saudi Arabia; 3 Infection Prevention and Control Department, King Abdulaziz Medical City, Riyadh, Saudi Arabia; 4 Saudi Centers for Disease Prevention and Control, Riyadh, Saudi Arabia; 5 Department of Medicine, King Abdulaziz Medical City, Riyadh, Saudi Arabia; 6 Pharmacy Department, King Abdulaziz Medical City, Riyadh, Saudi Arabia; 7 King Abdullah International Medical Research Center, Riyadh, Saudi Arabia

**Keywords:** Antimicrobial, Resistance, Appropriateness, Sepsis, Critical care response, Saudi Arabia

## Abstract

**Background:**

Most septic patients managed by critical care response teams (CCRT) are prescribed antimicrobials. Nevertheless, data evaluating their appropriateness are lacking both locally and internationally. The objective was to assess antimicrobial use among septic and non-septic patients managed by CCRT.

**Setting:**

Case-control design was used to compare septic (cases) and non-septic (controls) CCRT patients at tertiary care setting. The frequency of antimicrobial use was assessed before and after CCRT activation. The appropriateness of antimicrobial use was assessed at day four post-CCRT, based on standard recommendations, clinical assessment, and culture results.

**Main results:**

A total of 157 cases and 158 controls were included. The average age was 61.1 ± 20.4 years, and 54.6% were males, with minor differences between groups. The use of any antimicrobial was 100.0% in cases and 87.3% in controls (*p* < 0.001). The use of meropenem (68.2% versus 34.8%, p < 0.001) and vancomycin (56.7% versus 25.9%, p < 0.001) were markedly higher in cases than controls. The overall appropriateness was significantly lower in cases than controls (50.7% versus 59.6%, *p* = 0.047). Individual appropriateness was lowest with meropenem (16.7%) and imipenem (25.0%), and highest with piperacillin/tazobactam (87.1%) and colistin (78.3%). Only 48.5% of antimicrobials prescribed by CCRT were de-escalated by a primary team within four days. Individual appropriateness and de-escalations were not different between groups.

**Conclusions:**

Empiric use and inadequate de-escalation of broad-spectrum antimicrobials were major causes for inappropriate antimicrobial use in CCRT patients. Our findings highlight the necessity of urgent implementation of an antimicrobial stewardship program, including training and auditing of antimicrobial prescriptions.

## Introduction

Critical Care Response Teams (CCRT) at the hospitals of Ministry of National Guard Health Affairs (MNGHA) were designed to quickly assess and transfer, if needed, rapidly deteriorating ward patients to the intensive care unit [1, 2]. The ultimate goal of a CCRT is to prevent cardiopulmonary arrest, stabilize patients’ condition and help in optimizing the care provided by the primary team in general wards [2]. Previous data from MNGHA showed that the implementation of CCRT was successful in reducing the rates of cardiopulmonary arrest and total hospital mortality among ward patients [3].

The assertive resuscitation role of CCRT frequently involves the initiation of one or more antibiotics, especially in septic patients [4]. Irrespective of CCRT, a considerable proportion of antimicrobials used in the healthcare setting are considered inappropriate, both nationally (66%) [5] and internationally (14–79%) [6]. Additionally, the widespread use of antimicrobial agents especially broad-spectrum agents have been linked to the global emergence of antimicrobial resistance [7–9]. Moreover, the use of antimicrobials can be associated with a number of adverse events that can be life-threatening and require emergency care in some patients [10, 11].

Septic patients managed by CCRT are most likely to be initiated on one or more antimicrobials, frequently on an empiric basis. However, there is a lack of studies evaluating their appropriateness. Additionally, it is not clear whether the appropriateness among septic patients is similar to or different than other types of patients managed by CCRT. Moreover, the impact of inappropriate antimicrobial use on the reported benefits of CCRT has never been examined. Such information is exceptionally critical to promote, expand, and tailor future antimicrobial stewardship programs (ASP) in MNGHA or similar hospitals. This can potentially reduce the morbidity, cost, and resistance associated with inappropriate antimicrobial use among septic patients. The objective of this study was to compare the quantity and quality of antimicrobial use among CCRT patients during three identified stages of their management; before CCRT activation, during and on day four from activation of CCRT.

## Methods

### Setting

Two MNGHA hospitals in Riyadh, Saudi Arabia were utilized for this study. The facilities are tertiary care hospitals that serve over 750,000 Saudi National Guard soldiers, employees, and their families. Collectively, both sites include 1300-beds, 15 different intensive care units, and 50-inpatient wards.

### Populations

All patients above 15 years and managed by the CCRT during the study period were eligible to be included in the study.

### Study design

Case-control design was used to compare septic (cases) and non-septic (controls) CCRT patients, recruited prospectively for five months (January to May 2018). Case-control status was determined by the septic or none septic diagnosis based on a clinical algorithm known to and applied by the CCRT team and has been described before [3]. In the study, we considered patients identified as septic by the CCRT as “cases” and patients identified as none septic by the CCRT as “controls.” The case-control status was not blinded to the physicians, as the sepsis status is an integral part of case management. The study covered three-time stages; two days before CCRT activation, during CCRT activation (request of CCRT management), and after CCRT activation up to four days from CCRT activation (primary team responsibility). The term post-CCRT activation will be used in this manuscript to cover the last two stages.

### Sample size and sampling

It was estimated that a total 288 patients (144 cases and 144 controls) are required to detect the double-fold risk of inappropriate antimicrobial use between cases and controls, assuming that the inappropriateness in controls was 40%, at a significance level of 95% and a power of 80%. Assigning cases and controls was the responsibility of an infection control practitioner and a CCRT coordinator. Since local records reveal that sepsis patients constitute no more than 25% of all CCRT patients, all patients with sepsis were chosen as cases. For each case with sepsis, one control was chosen from the same week of CCRT patients using a systematic sampling technique.

### Data collection

In addition to the CCRT regular data collection form described before [3], a second structured data collection form was developed to evaluate the antimicrobial use and appropriateness. The form was initiated for any case or control once identified. Patient demographics, triggers of CCRT activation, and antimicrobial use information were recorded.

### Exposure definition

Sepsis was defined as per the third International Consensus Definitions for Sepsis and Septic Shock (Sepsis-3). Therefore, sepsis was defined as life-threatening organ dysfunction caused by a dysregulated host response to infection [12]. Septic shock was a subset of sepsis with circulatory and cellular/metabolic dysfunction associated with a higher risk of mortality [12]. Type of infection was determined as per the CDC National Healthcare Safety Network (NHSN) definitions for infections [13].

### Outcome definition

Use of antimicrobials was assessed by the number (and percentage) of patients using one or more antimicrobials during the three stages of the study relative to the total number of patients. Appropriateness of antimicrobial use was defined as the use of appropriate/first choice antimicrobial according to hospital antimicrobial guidelines, clinical assessment, culture results, and other relevant investigations. Hospital antimicrobial guidelines [14] were developed by the ASP working group of the hospital. The guidelines were created to reflect the Sanford [15] and John Hopkin’s [16] guidelines taking into consideration the local hospital formulary and microbiological data. Appropriateness was assessed for patients who were continued on antimicrobials through day four from CCRT activation. Day four was chosen to allow for a more objective decision based on culture and laboratory results. Appropriateness was assessed for the choice of agent, dose, duration, and route of the used antimicrobial for current indication. The appropriateness decision was reached by consensus of two board-certified infectious disease physicians. In case of disagreement, the opinion of a third infectious disease consultant physician was considered final. Antimicrobial adverse events included anaphylaxis, skin, hematologic, hepatic, renal, and other adverse events as defined by the Lexi-Comp, Inc. (Lexi-Drugs®) definitions [17]. CCRT outcomes included discharge home, stay at floor, ICU admission, and death. For ICU admission, it was defined as admission to ICU after CCRT activation but not at the time of the first CCRT assessment. If the patient died or discharged before day four, the outcome assessed was done according to the last available patient information during the current hospitalization.

### Statistical methods

Categorical variables were presented as frequencies and percentages. Continuous variables were presented as means and standard deviations (SD) or median and interquartile range (IQR), as appropriate. Non-matched case-control analysis was done. Demographic and clinical characteristics, antimicrobial use, and appropriateness were compared between cases and controls. Chi-squared test or Fisher’s exact test, as appropriate, were used to compare categorical variables. T-Test or Mann Whitney, as appropriate, were used to compare continuous variables. All *P*-values were two-tailed. A *p*-value < 0.05 was considered as significant. Statistical Package for the Social Sciences software (SPSS Version 25.0. Armonk, NY: IBM Corp) was used for all statistical analyses.

## Result

A total of 315 CCRT patients were included in the current analysis; 157 with sepsis (cases) and 158 without sepsis (controls). Among the 157 septic patients, 97 (61.8%) had a defined septic focus and 60 (38.2%) had no defined septic focus. Among those with defined septic focus, pneumonia (33.6%) was the main diagnosis followed by, bloodstream infection (21.6%), gastrointestinal infections (21.6%), and urinary tract infections (18.6%). The demographic and clinical characteristics of the patients by study group are shown in Table 1. For all patients, the average age was 61.1 ± 20.4 years and 54.6% of the patients were males. The average body mass index was 27.1 ± 7.8, with 33.3% of the patients classified as obese. Most of the patients were managed by a medical service (56.2%), followed by surgical (21.6%), and oncology services (14.0%). The most frequent triggers for CCRT activation were changes in respiratory rates (29.5%), hypotension (24.4%), changes in heart rate (21.0%), and drop of the Glasgow coma score (17.1%). The median (and IQR) hospital stay was 15 (10–30) days, of them the time before CCRT activation was 10 (2–33) days. Among septic patients, only 19 (12.3%) had CCRT activation within two days of admission (community-related). Compared to controls, cases had slightly lower age (*p* = 0.024), more likely to be oncology patients and less likely to be medical patients (*p* = 0.037), and had a longer time before CCRT activation (*p* < 0.001). For triggers, cases were more likely to be hypotensive (*p* < 0.001), have less drop of Glasgow coma score (*p* = 0.003), and represent less serious concern as perceived by the primary team (0.011).

**Table 1:** Demographic and clinical characteristics of CCRT patients by group

	Cases *N* = 157	Control *N* = 158	Total *N* = 315	p-value
Age (years)				
Mean ± SD	58.5 ± 21.4	63.6 ± 19.0	61.1 ± 20.4	0.024
< 50	52 (33.3%)	28 (17.7%)	80 (25.5%)	0.004
50–64	34 (21.8%)	57 (36.1%)	91 (29.0%)	
65–84	55 (35.3%)	57 (36.1%)	112 (35.7%)	
≥ 85	15 (9.6%)	16 (10.1%)	31 (9.9%)	
Gender				
Male	87 (55.4%)	85 (53.8%)	172 (54.6%)	0.773
Female	70 (44.6%)	73 (46.2%)	143 (45.4%)	
Body mass index				
Mean ± SD	27.1 ± 7.8	27.1 ± 7.7	27.1 ± 7.8	> 0.99
Non-obese	103 (65.6%)	107 (67.7%)	210 (66.7%)	0.69
Obese	54 (34.4%)	51 (32.3%)	105 (33.3%)	
Service				
Medical	79 (50.3%)	98 (62.0%)	177 (56.2%)	0.037
Surgical	36 (22.9%)	32 (20.3%)	68 (21.6%)	
Hepatobiliary	4 (2.5%)	6 (3.8%)	10 (3.2%)	
Oncology	27 (17.2%)	17 (10.8%)	44 (14.0%)	
Obstetrics and gynecology	0 (0.0%)	2 (1.3%)	2 (0.6%)	
Others	11 (7.0%)	3 (1.9%)	14 (4.4%)	
CCRT triggers				
Threatened airway	0 (0.0%)	1 (0.6%)	1 (0.3%)	> 0.99
Respiratory rate < 8 or > 30 per minute	45 (28.7%)	48 (30.4%)	93 (29.5%)	0.738
Oxygen saturation < 90%	21 (13.4%)	26 (16.5%)	47 (14.9%)	0.443
Systolic blood pressure < 90 mmHg	69 (43.9%)	8 (5.1%)	77 (24.4%)	< 0.001
Systolic blood pressure > 200 mmHg	1 (0.6%)	6 (3.8%)	7 (2.2%)	0.121
Heart rate < 40 or > 130 beats per minute	36 (22.9%)	30 (19.0%)	66 (21.0%)	0.39
Glasgow coma score drop ≥2	17 (10.8%)	37 (23.4%)	54 (17.1%)	0.003
Urine output ≤100 ml per 4 h	8 (5.1%)	6 (3.8%)	14 (4.4%)	0.576
Serious concern by treating team	2 (1.3%)	11 (7.0%)	13 (4.1%)	0.011
Others/unclear	15 (9.6%)	14 (8.9%)	29 (9.2%)	0.831
Important times (median & IQR)				
Length of stay (days)	14 (9–28)	16 (11–30)	15 (10–30)	0.468
Days before CCRT activation	14 (4–44)	7 (2–23)	10 (2–33)	< 0.001
Minutes from CCRT activation to AM order	33 (21–72)	46 (24–65)	36 (22–72)	0.243
Minutes from AM order to AM use	73 (37–115)	82 (48–150)	74 (38–120)	0.229

Abbreviation; CCRT, critical care response team; AM, antimicrobial; SD, standard deviation; IQR, interquartile range

The frequency of antimicrobial use by the group is shown in Table 2. There was significantly higher antimicrobial use in cases than controls at the three stages of the study. For example, the use of any antimicrobial during CCRT activation was 96.2% in cases and 65.2% in controls (*p* < 0.001). Similarly, the use of any antimicrobial at any stage was 100.0% in cases and 87.3% in controls (*p* < 0.001). For all patients at the three stages, the most frequently used antimicrobials were meropenem (51.4%), vancomycin (41.3%), piperacillin/tazobactam (28.6%). Further, meropenem (p < 0.001), vancomycin (p < 0.001), linezolid (*p* = 0.002), caspofungin (*p* = 0.001), and anidulafungin (*p* = 0.015) were more frequently used in cases than controls. Interestingly, a new antimicrobial agent was used post-CCRT activation in 45.4% of cases and 36.6% of controls (*p* = 0.010).

**Table 2:** Frequency of antimicrobial use among CCRT patients by group

	Cases *N* = 157	Control *N* = 158	Total *N* = 315	*p*-value
Overall antimicrobial use				
At any point	157 (100.0%)	138 (87.3%)	295 (93.7%)	< 0.001
Before CCRT activation	129 (82.2%)	111 (70.3%)	240 (76.2%)	0.013
During CCRT activation	151 (96.2%)	103 (65.2%)	254 (80.6%)	< 0.001
After CCRT activation	129 (82.2%)	107 (67.7%)	236 (74.9%)	0.003
Individual antimicrobial use at any point				
Meropenem	107 (68.2%)	55 (34.8%)	162 (51.4%)	< 0.001
Vancomycin	89 (56.7%)	41 (25.9%)	130 (41.3%)	< 0.001
Piperacillin-tazobactam	44 (28.0%)	46 (29.1%)	90 (28.6%)	0.831
Ceftriaxone	16 (10.2%)	22 (13.9%)	38 (12.1%)	0.311
Linezolid	21 (13.4%)	6 (3.8%)	27 (8.6%)	0.002
Colistin	13 (8.3%)	10 (6.3%)	23 (7.3%)	0.506
Caspofungin	18 (11.5%)	3 (1.9%)	21 (6.7%)	0.001
Anidulafungin	14 (8.9%)	4 (2.5%)	18 (5.7%)	0.015
Tigecycline	12 (7.6%)	5 (3.2%)	17 (5.4%)	0.079
Ciprofloxacin	6 (3.8%)	11 (7.0%)	17 (5.4%)	0.218
Imipenem	8 (5.1%)	6 (3.8%)	14 (4.4%)	0.576
Others	137 (87.3%)	85 (53.8%)	222 (70.5%)	< 0.001
Antimicrobial change after CCRT activation				
New prescription	254 (45.4%)	117 (36.6%)	371 (42.2%)	0.010
Continuation	305 (54.6%)	203 (63.4%)	508 (57.8%)	

**Fig. 1:**
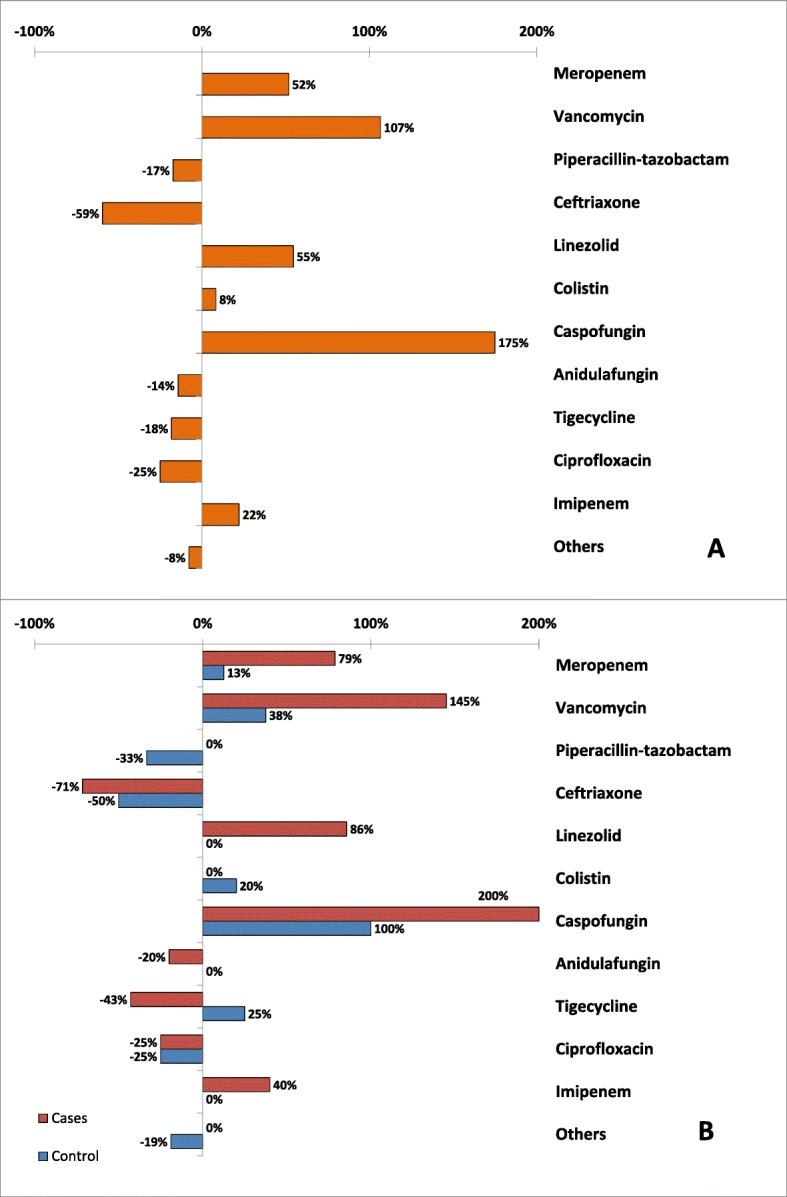
Percentage change of the frequency of antimicrobial use post-CCRT activation for all CCRT patients (**a**) and by group (**b**)

The details of changes in antimicrobial use post-CCRT activation are shown in Fig. 1. In all patients, the frequencies of use of caspofungin (175.0%), vancomycin (106.7%), linezolid (54.5%), and meropenem (51.9%) were considerably increased post-CCRT activation; all were prescribed mainly by the CCRT team except caspofungin, which was prescribed, mainly by the primary team. On the other hand, the frequency of use of ceftriaxone (− 59.4%), ciprofloxacin (− 25.0%), tigecycline (− 18.2%), and piperacillin/tazobactam (− 17.2%) were reduced post-CCRT activation. The first two were discontinued by both CCRT and the primary team while the last two were discontinued by CCRT only. With the exception tigecycline, which was increased in controls and reduced in cases, changes in the use for all antimicrobials were in the same direction in both cases and controls. Nevertheless, the post-CCRT increase of meropenem, vancomycin, linezolid, and caspofungin were higher in cases than control.

As shown in Table 3, obtaining cultures were more likely in cases than in controls (75.8% versus 51.9%, *p* < 0.001); and positive culture were more frequent in cases than controls (43.2% versus 28.0%, *p* = 0.029). In all patients, the most frequently retrieved pathogens were *Pseudomonas* spp. (16.9%), *Escherichia coli* (15.7%), *Candida* (10.8%), *Klebsiella* spp. (9.6%), and *Staphylococcus aureus* (8.4%). There were no significant differences in retrieved pathogens between cases and controls.

**Table 3:** Findings of culture done for CCRT patients by group

	Cases *N* = 157	Control *N* = 158	Total *N* = 315	*p*-value
Related culture done				
No	38 (24.2%)	76 (48.1%)	114 (36.2%)	< 0.001
Yes	119 (75.8%)	82 (51.9%)	201 (63.8%)	
Culture specimen				
Blood	106 (89.1%)	68 (82.9%)	174 (86.6%)	0.209
Urine	65 (54.6%)	47 (57.3%)	112 (55.7%)	0.705
Respiratory	21 (17.6%)	20 (24.4%)	41 (20.4%)	0.244
Wound	11 (9.2%)	3 (3.7%)	14 (7.0%)	0.126
Others	13 (10.9%)	3 (3.7%)	16 (8.0%)	0.061
Culture result				
No	67 (56.8%)	59 (72.0%)	126 (63.0%)	0.029
Yes	51 (43.2%)	23 (28.0%)	74 (37.0%)	
Organisms detected				
Total number	58	25	83	
Pseudomonas spp.	11 (19.0%)	3 (12.0%)	14 (16.9%)	0.666
*Escherichia coli*	9 (15.5%)	4 (16.0%)	13 (15.7%)	> 0.99
Candida	7 (12.1%)	2 (8.0%)	9 (10.8%)	0.905
Klebsiella spp.	6 (10.3%)	2 (8.0%)	8 (9.6%)	> 0.99
Staphylococcus aereus	5 (8.6%)	2 (8.0%)	7 (8.4%)	> 0.99
Enterococcus spp.	5 (8.6%)	1 (4.0%)	6 (7.2%)	0.822
MRSA	3 (5.2%)	3 (12.0%)	6 (7.2%)	0.504
Acinetobacter spp.	3 (5.2%)	1 (4.0%)	4 (4.8%)	> 0.99
Others	9 (15.5%)	7 (28.0%)	14 (19.3%)	0.308

Appropriateness of antimicrobial use at day four from CCRT activation is shown in Table 4. The overall appropriateness was significantly lower in cases (50.7%) than controls (59.6%, *p* = 0.047). On the other hand, dose and route components of appropriateness were close to 100% in both cases and controls. The overall appropriateness was highest with piperacillin/tazobactam (87.1%), colistin (78.3%) and ceftriaxone (73.3%); and lowest with meropenem (16.7%), imipenem (25.0%) and anidulafungin (33.3%). Less than half (48.5%) of antimicrobials prescribed by CCRT were de-escalated by the primary team within four days. The de-escalation rates for commonly used antimicrobials were 33.3% for meropenem, 54.8% for vancomycin, and 31.3% for piperacillin/tazobactam. None of individual antimicrobial appropriateness nor de-escalation were significantly different between cases and controls.

**Table 4:** Appropriateness of antimicrobial use at the fourth day of CCRT activation by group

	Cases *N* = 157	Control *N* = 158	Total *N* = 315	*p*-value
Appropriateness of all antimicrobials				
Overall	171 (50.7%)	118 (59.6%)	289 (54.0%)	0.047
Choice	180 (53.4%)	121 (61.1%)	301 (56.3%)	0.083
Dose	328 (97.6%)	196 (99.0%)	524 (98.1%)	0.433
Duration	179 (53.1%)	120 (60.6%)	299 (55.9%)	0.092
Route	336 (99.7%)	198 (100.0%)	534 (99.8%)	> 0.99
Overall appropriateness of individual antimicrobials				
Meropenem	14 (15.2%)	8 (20.0%)	22 (16.7%)	0.498
Vancomycin	33 (63.5%)	14 (58.3%)	47 (61.8%)	0.831
Piperacillin-tazobactam	29 (87.9%)	32 (86.5%)	61 (87.1%)	> 0.99
Caspofungin	6 (35.3%)	5 (71.4%)	11 (45.8%)	0.245
Colistin	9 (75.0%)	9 (81.8%)	18 (78.3%)	> 0.99
Linezolid	9 (52.9%)	4 (80.0%)	13 (59.1%)	0.587
Tigecycline	6 (46.2%)	3 (75.0%)	9 (52.9%)	0.671
Anidulafungin	5 (50.0%)	0 (0.0%)	5 (33.3%)	0.168
Ceftriaxone	4 (66.7%)	7 (77.8%)	11 (73.3%)	> 0.99
Ciprofloxacin	5 (83.3%)	5 (55.6%)	10 (66.7%)	0.587
Imipenem	1 (25.0%)	2 (25.0%)	3 (25.0%)	> 0.99
Others	50 (66.7%)	29 (74.4%)	79 (69.3%)	0.398
Post-CCRT de-escalation of individual antimicrobials by primary team				
Meropenem	28 (33.3%)	12 (33.3%)	40 (33.3%)	> 0.99
Vancomycin	39 (54.9%)	12 (54.5%)	51 (54.8%)	0.975
Piperacillin-tazobactam	7 (25.0%)	8 (40.0%)	15 (31.3%)	0.269
Caspofungin	3 (33.3%)	2 (100.0%)	5 (45.5%)	0.182
Colistin	3 (42.9%)	3 (50.0%)	6 (46.2%)	> 0.99
Linezolid	5 (38.5%)	0 (0.0%)	5 (29.4%)	0.261
Tigecycline	1 (25.0%)	3 (60.0%)	4 (44.4%)	0.524
Anidulafungin	2 (50.0%)	1 (50.0%)	3 (50.0%)	> 0.99
Ceftriaxone	2 (50.0%)	7 (77.8%)	9 (69.2%)	0.530
Ciprofloxacin	1 (33.3%)	2 (33.3%)	3 (33.3%)	> 0.99
Imipenem	3 (42.9%)	4 (100.0%)	7 (63.6%)	0.194
Others	46 (70.8%)	26 (66.7%)	72 (69.2%)	0.867
Overall	140 (46.8%)	80 (51.6%)	220 (48.5%)	0.333

As shown in Table 5, more cases were likely to be admitted to ICU than controls (43.9% versus 28.5%, *p* = 0.004). There were no differences in mortality (4.5% versus 1.3%, *p* = 0.104) or developing antimicrobial adverse events (31.8% versus 27.2%, *p* = 0.270) between cases and controls. The frequency of adverse events is detailed in Table 5.

**Table 5:** CCRT outcomes and antimicrobial adverse events by the fourth day of CCRT activation by group

	Cases *N* = 157	Control *N* = 158	Total *N* = 315	*p*-value
Outcome				
Stay at floor	77 (49.0%)	94 (59.5%)	171 (54.3%)	0.063
Admission to intensive care unit	69 (43.9%)	45 (28.5%)	114 (36.2%)	0.004
Death	7 (4.5%)	2 (1.3%)	9 (2.9%)	0.104
Discharge home	0 (0.0%)	2 (1.3%)	2 (0.6%)	0.498
Others	0 (0.0%)	2 (1.3%)	2 (0.6%)	0.498
Antimicrobial adverse events				
None	107 (68.2%)	115 (72.8%)	222 (70.5%)	0.270
One	36 (22.9%)	36 (22.8%)	72 (22.9%)	
Two or more	14 (8.9%)	7 (4.4%)	21 (6.7%)	
Types of antimicrobial adverse events				
Renal	21 (13.4%)	18 (11.4%)	39 (12.4%)	0.593
Hematologic	17 (10.8%)	19 (12.0%)	36 (11.4%)	0.738
Gastrointestinal	17 (10.8%)	7 (4.4%)	24 (7.6%)	0.032
Hepatic	8 (5.1%)	6 (3.8%)	14 (4.4%)	0.576
Skin	3 (1.9%)	1 (0.6%)	4 (1.3%)	0.371
Anaphylaxis	0 (0.0%)	1 (0.6%)	1 (0.3%)	> 0.99

## Discussion

The current study examined in a case-control design the pattern of antimicrobial use among inpatients with or without sepsis managed by the CCRT in a tertiary care setting.

The main finding was the high level (46%) of inappropriate antimicrobial use, largely caused by inadequate de-escalation of broad-spectrum antimicrobials by the primary care teams within four days from CCRT activation. Even though we could not identify similar published studies looking into CCRT patients, inappropriate use is known to be very frequent among hospitalized patients especially those admitted to intensive care units [6]. The significantly higher inappropriate antimicrobial use among septic patients may reflect the concern, among the providers in our centers, of resistant pathogens as an etiology for sepsis [18, 19]. There is some legitimacy for these concerns since recent local and national data have highlighted significant emergence of resistance, especially to carbapenems [20–23]. For example, we had reported in our hospital the first national carbapenem-resistant Klebsiella *pneumonia* (CRKP) outbreak that took place in an ICU setting [24].

The current study showed a heavy antimicrobial use in both cases and controls. While the heavy antimicrobial use in patients with sepsis was expected, the still heavy antimicrobial use in the patients without sepsis after CCRT activation may be related to the limited de-escalation rate after CCRT activation (51.6%), the high rate of antimicrobial use before CCRT activation (70.3%), and the considerable diagnosis of infection at admission (20.3%).

Assessment of antimicrobial appropriateness was done by two board-certified infectious disease physicians to allow for fair auditing by qualified assessors outside the primary and CCRT teams. It was done at day four from CCRT activation to allow the results of cultures and other laboratory tests to be released and the clinical status of the patient to be more clear, so as to reach a more objective decision about tailoring down therapy [25]. The infectious disease physicians were not allowed to interfere with prescriptions (during the study through the day four from CCRT activation), so as to accurately assess the burden of the problem and create recommendations that reflect currently implemented practices.

The fact that one-fourth of cases and one-half of controls never underwent appropriate investigation in the form of a culture may indicate the importance of introducing an ASP that ensures the proper diagnostics are in place and implemented. In the absence of proper cultures, the de-escalation process becomes more challenging. Other locally perceived challenges for implementing timely de-escalation include: limited ASP team members available for guidance and auditing, lack of confidence of the primary team caring for critically ill patients to change previously prescribed broad-spectrum antimicrobials, poor knowledge on mechanisms of resistance, and underestimation of the impact of misuse of antibiotics on the emergence of resistant bacterial strains [26].

The experiences of ASP programs in other institutions have shown the impact on prescription patterns; and at the same time has not allowed for higher mortality rates among their patients [27, 28]. The current study is considered one of the recent ASP efforts in our centers that aimed to quantify the burden of antimicrobial misuse in a special population with identified high levels of use. The ASP team was able to provide, through this study, evidence to the primary teams on the burden of misuse and highlight the need for their engagement and collaboration with the ASP team members. It also highlighted to the leadership the importance of sustaining the support for the ASP teams, and especially to focus on educational activities targeting both primary and CCRT teams. Furthermore, it ingrained our belief in the importance of providing prescriber-feedback on their use of antibiotics as a major intervention to improve appropriateness [29, 30]. The main essences are to identify barriers, consider resources, and tailored ASP activities [29, 30].

The current findings did not reveal significant differences between cases and controls with regards to mortality or antimicrobial adverse events. And the findings cannot be used to assess the CCRT activities due to the lack of a control group not managed by CCRT [3]. The higher ICU admission rates among cases were probably driven by the rapidly deteriorating conditions of septic patients. However, this cannot be directly linked to the level of antimicrobial appropriateness using the current study design.

### Study limitations

Our findings need to be interpreted cautiously as it represents two sites of one institution and generalization of results may not be applicable to many hospitals. The assessment timing on day four may have over-estimated the inappropriateness level as the medications that were discontinued before day four (assumed to have a higher level of appropriateness) were not included in the assessment. However, day four was chosen to allow the time needed to establish the final clinical diagnosis and to have culture results available [25]. The cases and controls were not matched on factors such as demographics and morbidity risks for logistic reasons. However, this should not have any significant effect on the appropriateness, which was individually assessed. The case-control status was not blinded to the physicians, as the sepsis status is an integral part of the case management. However, the controls were chosen using a systematic sampling technique done by and known only by two study members. Additionally, we believe that the non-blinding had no impact on the study findings as the high antimicrobial use before joining the study was maintained after joining the study (at CCRT activation). Furthermore, the appropriateness and de-escalation of individual antimicrobials did not show significant differences between groups.

## Conclusions

Empiric use and inadequate de-escalation of broad-spectrum antimicrobials were probably responsible for heavy and largely inappropriate antimicrobial use among CCRT patients, especially septic ones. There were no significant differences in mortality or antimicrobial adverse events between groups. Our findings highlight the necessity of urgent implementation of an antimicrobial stewardship program, tailored to our patient needs. This should include training and auditing of antimicrobial prescriptions. Future studies can focus on the impact of such ASP strategies on CCRT outcomes.

## Data Availability

The datasets used and/or analysed during the current study are available from the corresponding author on reasonable request.
